# Digesta and Plasma Metabolomics of Rainbow Trout Strains with Varied Tolerance of Plant-Based Diets Highlights Potential for Non-Lethal Assessments of Enteritis Development

**DOI:** 10.3390/metabo11090590

**Published:** 2021-09-01

**Authors:** Mariana Palma, Jacob W. Bledsoe, Ludgero C. Tavares, Nicholas Romano, Brian C. Small, Ivan Viegas, Ken Overturf

**Affiliations:** 1 Centre for Functional Ecology, Department of Life Sciences, University of Coimbra, 3000-456 Coimbra, Portugal; mpalma@uc.pt; 2 ARS-USDA, Hagerman Fish Culture Experiment Station, Hagerman, ID 83332, USA; bledsoe@uidaho.edu (J.W.B.); ken.overturf@usda.gov (K.O.); 3 CIVG—Vasco da Gama Research Center, University School Vasco da Gama—EUVG, 3020-210 Coimbra, Portugal; ludgero.tavares@euvg.pt; 4 Center for Neuroscience and Cell Biology, University of Coimbra, 3004-517 Coimbra, Portugal; 5 Center of Excellence in Aquaculture & Fisheries Center, University of Arkansas at Pine Bluff, Pine Bluff, AR 71601, USA; romanon@uapb.edu; 6 Aquaculture Research Institute, Hagerman Fish Culture Experiment Station, University of Idaho, Hagerman, ID 83332, USA; bcsmall@uidaho.edu

**Keywords:** aquaculture, NMR metabolomics, plant-based aquafeed, rainbow trout, soybean meal, animal selection

## Abstract

The replacement of fishmeal in aquafeeds is essential to the sustainability of aquaculture. Besides the procurement of alternative protein sources, fish can also be selected for better performance on plant-based alternative diets. Rainbow trout (*Oncorhynchus mykiss*) is one such species in which the strain ARS-*Sel* has been selected for higher growth and enhanced utilization when fed soy-based diets. The aim of this study was to compare fish growth and plasma and digesta metabolomes between ARS-*Sel* and two commercial strains (CS-1 and CS-2), when fed plant-protein (PM) and fishmeal-based (FM) diets, and to correlate them with the onset of enteritis. An NMR-metabolomics approach was taken to assess plasma and digesta metabolite profiles. Diet and strain showed significant effects on fish growth, with the ARS-*Sel* fish receiving the PM diet reaching the highest final weight at sampling. Multivariate analysis revealed differences between plasma and digesta metabolite profiles of ARS-*Sel* and CS (CS-1 considered together with CS-2) PM-fed groups in the early stages of enteritis development, which was confirmed by intestinal histology. As reported in previous studies, the ARS-*Sel* strain performed better than the commercial strains when fed the PM diet. Our findings also suggest that metabolomic profiles of plasma and digesta, samples of which can be obtained through non-lethal methods, offer valuable insight in monitoring the occurrence of enteritis in carnivorous aquaculture species due to plant-based diets.

## 1. Introduction

Aquaculture continues to be the most rapidly expanding form of animal agriculture. While finfish aquaculture continues to develop and grow, its growth has been limited partially due to the reliance of fishmeal as a primary protein source in aquaculture feeds. Though some species are still dependent on fishmeal, a concerted effort to reduce the use of fishmeal should promote aquaculture sustainability by decreasing dependence on limited wild stocks and by reducing productions costs [1,2]. Plant derived proteins from several sources (e.g., soybean, barley, wheat, corn, peas, rapeseed, linseed, and white lupin) have been extensively tested over the last several years as protein sources for carnivorous fish, with variable results [3,4,5,6]. Several serious issues have arisen when replacing fishmeal with plant-derived proteins in aquafeeds, including reduced growth, increased mortality and effluent waste, intestinal inflammation, and chronic immune stimulation [7,8,9,10].

Besides the quest for alternative plant-protein sources and for additives that could attenuate the effects of the dietary plant-protein, fish species are also being nutritionally programmed [11] and even selected towards improved physiological and zootechnical performances, when fed plant-based diets. Selection in aquaculture species has mainly been driven towards the main production traits such as growth, disease resistance, feed intake, feed efficiency [12,13], and fillet features like yield [14], and fat content [15,16]. The advances brought by this type of strategy underpin its feasibility to generate cohorts of individuals potentially more adapted to feeds with increased plant-protein contents.

Rainbow trout (*Oncorhynchus mykiss*) is one of the major species produced in freshwater aquaculture worldwide, with production and demand expected to increase in the future [2]. With an extensive body of literature on its nutrition and farming process, this species has been ideally suited for use as an experimental model in the context of improving plant-protein utilization in carnivorous fish. In fact, over the last decade, our group has utilized family-based mass selection of rainbow trout over several generations to generate a strain, denoted here as ARS_*Sel*, that shows increased growth performance when fed soy-based diets, resistance to soy-induced enteritis, and improved growth performance and non-specific pathogen resistance [17,18,19,20,21,22]. As previously noted, inflammation of the intestine, also termed enteritis, has been the predominant concern when plant derived protein products are incorporated at high levels in aquafeeds, particularly for carnivorous species, as it is thought to contribute to reduced growth, reduced feed efficiency, and increased mortality. To date, traditional techniques for evaluating soy-induced enteritis involve histological and transcriptional markers which require lethal sampling methods and time-consuming protocols.

Nuclear Magnetic Resonance (NMR)-metabolomics has been used to assess the metabolic utilization of alternative dietary ingredients in several fish species. NMR spectroscopy has the advantage of being quantitative and qualitative, highly reproducible and able to detect compounds within a wide range of physiochemical properties, which highlight its usefulness in metabolic studies. If combined with multivariate data analyses such as principal component analysis (PCA) and partial least-squares analysis (PLS), NMR-metabolomics provides important insights into multiple physiological and metabolic alterations caused by different ingredients. This methodology has been applied to profile alterations from dietary treatments in various biological tissues like muscle or liver in several fish species [5,23,24,25], including in rainbow trout [26,27]. NMR-based metabolomics has also been able to characterize plant-based feed formulations for rainbow trout [28].

In order to diagnose the onset and progression of enteritis, serum and digesta could be sampled in a non-lethal manner, allowing for a continued assessment of any alteration to the intestinal absorption of nutrients. NMR-based plasma metabolomics has been used to monitor dietary imbalances [29] including those provoked by the use of soy-based protein products [30], while digesta metabolomics was used as a tool to evaluate changes in fish gut health [31], acting as a proxy for the evaluation of digestive/absorptive processes.

The aim of the study was to determine if a non-lethal sampling method could be developed to monitor enteritis advancement in fish when fed plant-based feeds. For this study, we utilized three strains of trout, our selected strain of rainbow trout that has been shown to be resistant to the development of enteritis when reared on a high soybean meal all plant protein diet (ARS-*Sel*) and two strains of commercial trout that have previously shown susceptibility to the development of enteritis when fed the same diet (CS-1 and CS-2).

## 2. Results

### 2.1. Fish Growth

Fish weights were tested separately by two-way ANOVA (strain by diet) at each sampling period (Table 1). At the first sampling time point, both trout strains and the strain by diet interaction term were found to significantly influence fish weight. A Tukey’s post-hoc test of strain effects indicated CS-2 weighed significantly less than both ARS-*Sel* and CS-1 (Table 1). At the conclusion of the trial, two-way ANOVA indicated only a significant effect of diet on fish weight, with the fish receiving the PM diet showing reduced weight compared to those on the FM diet (Table 1).

### 2.2. Intestinal Histology

**Figure 1:**
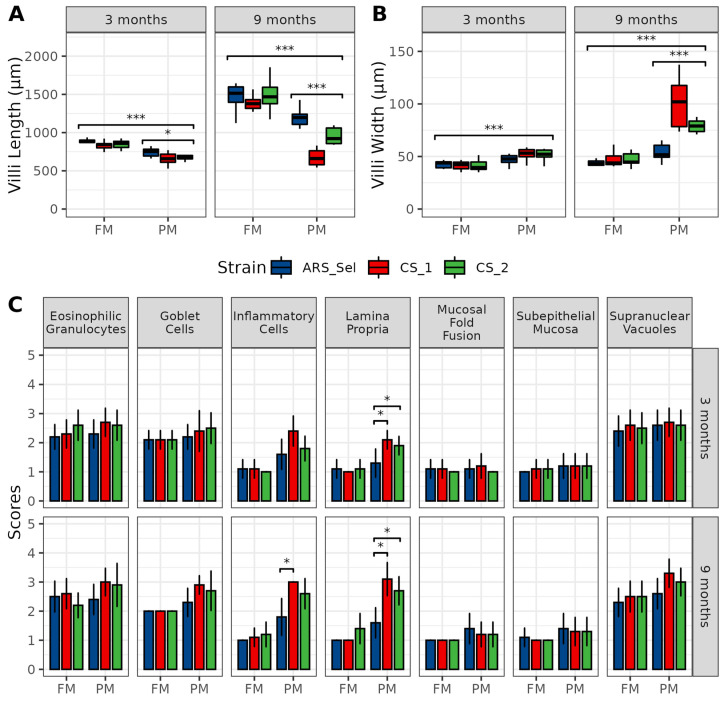
Results from histological analyses of distal intestinal samples from one selected (ARS-*Sel*) and two commercial strains (CS-1 & CS-2) of rainbow trout fed either fishmeal- or plantmeal-based diets at 3 and 9 months. Continuous data on intestinal villi length (**A**) and width (**B**) were quantified by imaging software and tested by Kruskal–Wallis to detected difference between diets across all strains, as well as strain differences within diets. Ordinal scoring data (ranked 1–5) for other histological metrics were analyzed using a paired Wilcoxon rank-sum test comparing both commercial strains to the ARS_Sel strain within diet and timepoint, with a Holm–Bonferroni multiple comparisons correction (**C**). * *p* ≤ 0.05, *** *p* ≤ 0.001.

At both timepoint 1 and 2 (3 months and 9 months), fish receiving the PM diet had significantly shorter (*p* < 0.001) and wider villi (*p* < 0.001) than fish receiving the FM diet, though these dietary differences were more exaggerated at timepoint 2 (9 months on diet) (Figure 1A,B). Additionally, strain effects were observed at both timepoints for villi length and at 9 months for villi width, with ARS_*Sel* exhibiting the healthier phenotype relative to the CS strains. Pairwise rank-sum tests indicated no significant differences in ordinal scored histological parameters when fish were fed the FM diet. When fed the PM diet, the ARS_*Sel* fish showed significantly healthier lamina propria compared to both strains at both timepoints (*p* ≤ 0.044), while also having a reduced level of inflammatory cells compared to CS_1 (*p* = 0.021) at the conclusion of the study (Figure 1C).

### 2.3. Plasma and Digesta Metabolome Analysis

In the plasma samples, 36 metabolites were identified and quantified by resorting to the automated Bayesil online system (Supplementary Table S3). Regarding digesta, two samples from the ARS-*Sel*_PM group, one sample from CS-1_PM, and one sample from the CS-1_FM group were removed from the study since their NMR spectra did not meet the required quality to allow rigorous peak identification and quantification. In the digesta samples, 21 metabolites were manually identified (Supplementary Table S4).

As a first approach to the data, the two commercial lines (CS-1 and CS-2) were compared by diets (PM and FM), to evaluate general group clustering and the similarity between the correspondent metabolite profiles. The PCA models for both plasma and digesta showed substantial overlap of groups CS-1 and CS-2 in both comparisons by diet (plasma: Supplementary Figure S1A,C; digesta: Supplementary Figure S2A,C). In all pairwise comparisons, the PLS models were not validated by the permutation testing (plasma: Supplementary Figure S1B,D; digesta: Supplementary Figure S2B,D), meaning the metabolite profiles of both strains were similar within the same diet and tissue. Upon this result, the groups from the two commercial lines (CS-1 and CS-2) were joined by diet and considered as a single group, hereafter designated as CS (CS_FM and CS_PM). The new empirical groups were then subjected to multivariate analyses considering the pairwise comparisons between the fish strains.

**Figure 2:**
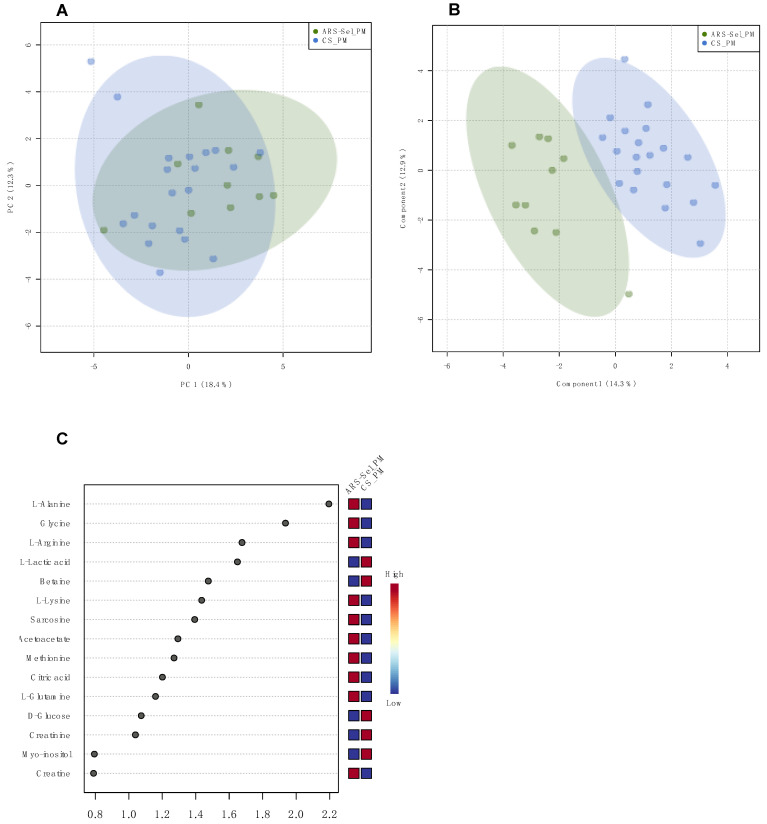
Multivariate analysis of groups ARS-*Sel*_PM and CS_PM in plasma: (**A**) PCA scores plot; (**B**) PLS scores plot, model validated (NC = 2; R^2^ = 0.826; Q^2^ = 0.543; 1000 permutations: *p* = 0.025); (**C**) VIP of the PLS scores plot model.

In plasma, the PCA model of the pairwise comparison between groups fed the PM diet revealed the groups are generally superimposed (Figure 2A). The PLS model was able to separate the two groups along the diagonal between component 1 and component 2 (Figure 2B). Permutation testing showed the model to be valid (*p* = 0.025) and 13 metabolites showed variable importance in projection (VIP) scores ≥ 1 (Figure 2C).

**Figure 3:**
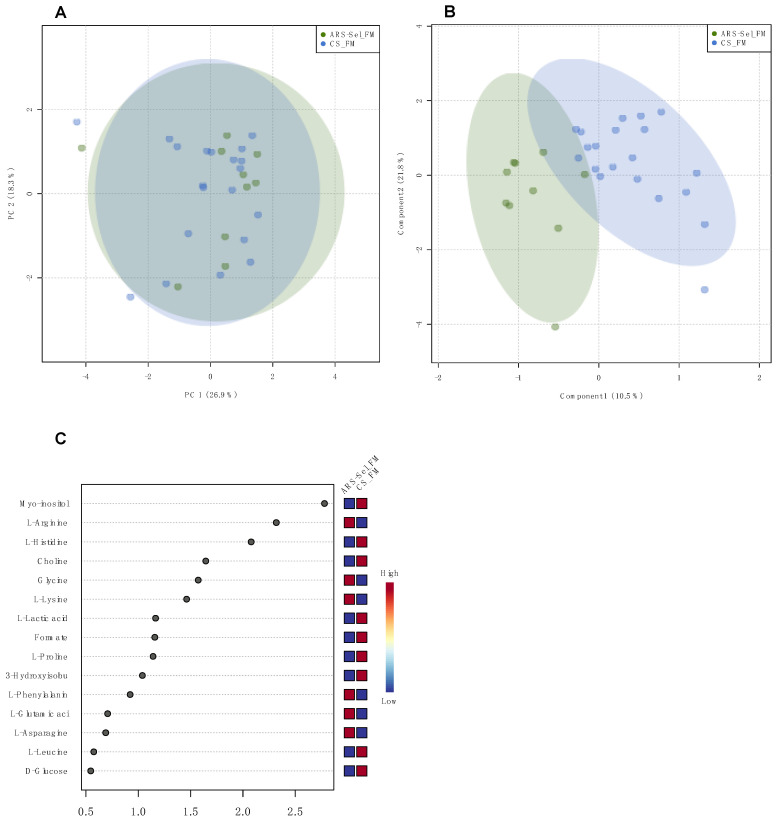
Multivariate analysis of groups ARS-*Sel*_FM and CS_FM in plasma: (**A**) PCA scores plot; (**B**) PLS scores plot, model validated (NC = 3; R^2^ = 0.841; Q^2^ = 0.361; 1000 permutations: *p* = 0.04); (**C**) VIP of the PLS scores plot model.

Regarding the pairwise comparison between the FM-fed groups, the PCA model showed a complete superimposition of the groups (Figure 3A). The PLS model revealed the groups are roughly separated and the model was validated (*p* = 0.04), with 10 metabolites showing a VIP score ≥ 1 (Figure 3B,C).

**Figure 4:**
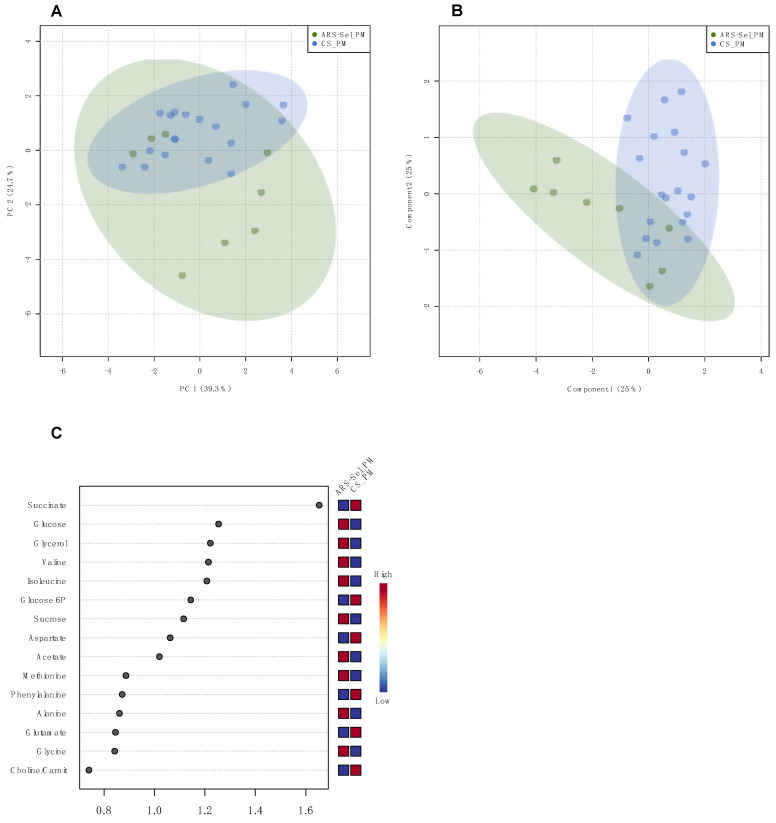
Multivariate analysis of groups ARS-*Sel*_PM and CS_PM in digesta: (**A**) PCA scores plot; (**B**) PLS scores plot, model validated (NC = 1; R^2^ = 0.445; Q^2^ = 0.176; 1000 permutations: *p* = 0.042); (**C**) VIP of the PLS scores plot model.

Regarding the multivariate analysis of the digesta samples, the PCA pairwise comparison of the groups fed the PM diet revealed both groups were clustered together (Figure 4A). In this model, the ARS-*Sel*_PM group has a higher dispersion of the samples, denoting a higher variation in the metabolome profiles within these individuals. The PLS scores plot presented a separation between the groups along a diagonal between component 1 and component 2, which contributed almost equally (25% each) to explain variations between clusters. Since this PLS model was validated by permutation testing (*p* = 0.042), it was possible to analyze the metabolites assigned as VIP, which showed higher contribution to the separation between strains (Figure 4B,C).

The pairwise multivariate comparison of the groups fed the FM diet revealed the strains to be mostly superimposed in both PCA and PLS models (Supplementary Figure S3), suggesting the metabolite profiles of the strains are similar. Since the PLS model was not validated, it was not possible to assess VIP scores. The results of the models for both tissues are summarized in Table 2.

## 3. Discussion

Here we aimed to develop a physiological assay for evaluating dietary performance of feeds with a high inclusion of alternative plant proteins, with the hopes of not only further characterizing ARS_*Sel* strain performance, but also to aid in the prediction of the development of soy-induced enteritis. Our approach compared the growth performance and NMR metabolome profiles of rainbow trout selected for plant-based diet utilization to commercial controls using biological materials which can be collected using non-lethal sampling techniques (i.e., blood plasma and feces), while confirming phenotypic differences in dietary response using traditional histological analysis.

By the end of our feeding trial, diet showed a significant impact on growth overall, with the FM-fed fish showing significantly higher final weights, as is typically observed. In the middle of the experiment, the ARS_*Sel* fish receiving the PM diet had the highest average weight. Furthermore, when controlling for diet and strain effects, pairwise comparisons of interaction groups showed the ARS-*Sel* fish fed the PM diet significantly outperformed the CS_2 fish fed both the PM and FM diet, while also mathematically outperforming CS_1 fish receiving the PM diet. These results suggest the selected strain is more amendable to diets with high plant-protein contents than the commercial strains, allowing for the maintenance of optimum growth. Similar growth performances have repeatedly been observed in studies comparing the growth performance of our selected strain to that of industry control strains while being fed sustainable high plant-protein diets [17,18,20].

As presented here and in previous studies [17,18,19,20,21,22], selection programs for the enhanced utilization of diets with a high inclusion of plant proteins can be a feasible approach to reducing aquaculture dependence on fishmeal by increasing the tolerance to sustainable plant proteins of carnivorous finfish. The development of lines of fish which utilize plant proteins more efficiently is beneficial for practical reasons, namely improving the environmental and economic sustainability of aquaculture feed sources. Furthermore, such lines can also serve as valuable research models for evaluating physiological adaptations utilized to maintain optimal growth and resistance to dietary perturbations not only in aquaculture species, but more broadly as well. The soybean-meal induced enteritis developed in teleost fishes, such as that seen in the commercial strains evaluated in this study, is similar in phenotype to ulcerative colitis or Crohn’s disease which afflict humans [32]. A characterization of the metabolic and physiologic adaptations observed in the ARS_*Sel* fish which showed resistance to soybean-meal induced enteritis could aid in the identification of treatments to alleviate intestinal inflammation in other species.

Our first approach to the metabolomics analysis revealed that the two commercial lines had similar final metabolite profiles when fed the same diet (PM or FM), in both the plasma and the digesta. These results are indicative of similar metabolic responses and allowed us to group samples from the two commercial strains by diet, increasing the number of data points and the robustness of the consequent multivariate models. When the ARS-*Sel* strain was compared with the merged CS group by diet, a complete group separation was observed between the two pairwise comparisons (ARS-*Sel*_PM vs. CS_PM, and ARS-*Sel*_FM vs. CS_FM) in the plasma, whereas in the digesta, only the pairwise comparison between ARS-*Sel*_PM vs. CS_PM revealed group separation.

In plasma, when the select and commercial strains were compared, groups were separated by valid PLS models in both the FM and PM diets, which allowed for the assessment of the VIP scores for metabolites involved in dietary metabolome variation. A majority of the metabolites responsible for the separation in strains fed the PM diet were amino acids, with all of them showing lower circulating concentration in the CS group. Previous studies with the same selected strain fed diets with different amino acid composition showed the ratio of dietary amino acids to closely correspond with plasma amino acid concentrations [21]. However, dietary contents of the amino acids highlighted by plasma metabolome analysis (alanine, glycine, arginine, lysine, methionine, and glutamine) were similar across dietary treatments, and even so, all statistical comparisons were between different strains receiving the same diets. As such, the variation in plasma metabolites seems to be attributed to the selected strain utilizing nutrients from the PM diet more efficiently than the commercial strains, which is in line with previous results that showed that, when fed the plant-based diet, the ARS_*Sel* strain demonstrated higher protein digestibility and absorption than a commercial trout strain [21]. The putative increased protein retention efficiency and subsequent growth rate justify the higher final fish weight of individuals from ARS-*Sel* at the end of the experiment. This imbalance in the amino acid levels between the two strains could be a direct result of the early development of enteritis that critically affects the intestinal absorption capacity and nutrient availability over time [18]. Different absorption dynamics between strains could led to different amino acid availability during protein synthesis [21] and therefore could directly influence muscle growth.

Changes were observed in plasma levels of glycine in pairwise comparisons of strains in both the FM- and PM-fed groups. Glycine is a key metabolite, and among other physiological functions, it can be directly used to produce energy or to synthesize proteins [33]. Regardless of the diet, glycine was observed in higher concentrations in the ARS-*Sel* group when compared with the CS strains. At the time of sampling, both strain and strain-by-diet interactions significantly influenced fish weight, which was highest in the ARS-*Sel*_PM group (Table 1), suggesting the increased glycine was utilized for protein synthesis pathways in the selected fish. Moreover, the expression of genes related to glycine-serine-threonine metabolism was also found to be altered during the onset of enteritis in rainbow trout fed formulated diets high in soybean meal [19]. In that same study, variations in gene expression related to glycolysis-gluconeogenesis pathways, the alanine-aspartate-glutamate metabolism, cysteine-methionine metabolism, and the TCA cycle were also observed during enteritis progression in fish fed plant-based diets [19]. Notably, in the present study, some of the intermediates of these pathways such as alanine, methionine, and citrate were highlighted by VIP scores in plasma and varied by fish strain when fed either diet. Methionine, an important methyl donor in several biosynthetic processes, is an essential amino acid to fish, and is commonly supplemented in plant-based diets to meet dietary requirements for growth [34,35,36]. VIP scores indicated methionine was an important metabolite involved in the separation of the plasma metabolomes of the ARS-*Sel* and CS strains fed the PM diet, with consistently higher methionine concentrations in individuals from the selected strain.

Comparisons of the digesta metabolomes between the selected and commercial trout strains showed no separation when fish received the FM diet, yet significant separation was observed for the PM-fed groups. Metabolites identified by VIP scores in the PM digesta comparisons suggest differences in energy metabolism between the strains may be affected by the divergent assimilation of the molecules involved in glucose metabolism and the TCA cycle, such as glucose, glucose-6-phosphate, succinate, isoleucine, valine, and acetate. A similar study used ultra-performance liquid chromatography-mass spectrometry to compare the digesta metabolome of a single cohort of hybrid grouper fed either a FM control or a diet containing soybean meal to identify 17 metabolites that could potentially serve as biomarkers of enteritis [37]. None of the potential biomarkers identified in that study were highlighted as differential in our study; however, our analyses compared metabolomes of strains with varied performance within diet type (FM and PM), whereas Zhang et al. compared a single cohort across diets [37]. These discrepancies in study design easily explain the differences in our findings. Yet, when taken together, these results suggest digesta metabolomics is a useful technique for studying plant diet-induced enteritis in fish. Although, one must consider that the metabolite profile of digesta samples comprises an inherent contribution of metabolites derived, not only directly from the digestion process, but also from the metabolic activities of the gut microbiota. These combined responses complicate the interpretation of the data, as they can reflect a dynamic adaptive response to the experimental diets on the fish metabolism and the microbiota community. Significant variations in the gut microbiota composition, namely in the abundance of some bacterial genera responsible for a diverse range of nutrient conversion after changes in diet composition were previously observed in fish species [38] such as the Atlantic salmon (*Salmo salar*) [39], rainbow trout [40], and European seabass [31]. Furthermore, work from our group has shown significant shifts in gut microbiota communities according to diet (FM vs. PM) and strain (ARS-*Sel* vs. CS_2) [20], potentially explaining some differences in digesta metabolomes seen here between the ARS-*Sel*_PM and CS_PM trout.

In general, the observed results seem indicative of a similar response across fish strains when fed the FM diet, but a divergent response when fed the PM diet, which is likely attributed to the strain-selection process of the ARS-*Sel* towards greater performance on plant-based diets. Taken together, these results confirm that selection can promote an integrated adaptation to alternative, more sustainable, plant-based diets among aquaculture species.

## 4. Material and Methods

### 4.1. Animal Experiment

The trial was conducted at the facilities of the Hagerman Fish Culture Experiment Station of the University of Idaho Aquaculture Research Institute, USA. For this experiment, 140 rainbow trout (*Oncorhynchus mykiss*) from each of three strains were used: ARS-*Sel*: the select strain known for superior growth performance on plant-based diets, resistance to soy induced enteritis, and disease resistance [17,18,19,20,21,22], Commercial Strain 1 (CS-1): a commercial strain selected for growth and pathogen resistance utilized within the aquaculture industry in the western United States, and CS-2: a commercially available strain from a large international egg supplier. All standard operating procedures, animal husbandry, and sampling techniques utilized in this study were pre-approved by the University of Idaho’s Institutional Animal Care and Use Committee under IACUC protocol #2019-16 “Strain and Diet Effects on the Intestinal Metabolome.” All research was conducted by IACUC-trained and approved researchers.

Female-only eggs from multiple families were gathered from each of the respective strains. Eggs from each strain were hatched in separate Heath trays supplied with the same spring-fed, flow-through water source (15 °C). At 8 weeks post hatch (~5 g body weight), fish from each strain were split into two 142 L tanks to be fed either a plant-protein based diet (PM) or a traditional fishmeal-based diet (FM) (Table 3). The detailed amino acid composition of the two diets is described in Supplementary Table S1. Fish were stocked at equal densities with feed administered to satiation three times daily, six days a week for eight weeks and then fed twice daily for the remainder of the experiment. Experimental groups were reared in separate tanks supplied with the same flow-through water (15 °C; 45 L min^−1^). Sampling occurred at five months post hatch (three months after initiation of the FM and PM diet trial), with samples collected three hours post-prandial from ten fish in each experimental group using a time-staggered, randomly assigned tank sampling order. Fish were euthanized by an overdose with tricaine-methanesulfonate following AVMA guidelines [41], measured and weighed. Whole blood samples were collected by caudal venipuncture using a heparinized syringe and held on ice until further processed. Fecal contents were collected from the distal intestine following dissection and removal of the intestinal tract. Additionally, sections of the distal intestine were collected and fixed in 10% formalin for histological evaluations of enteritis development using traditional eosin and hematoxylin staining techniques. Following the initial sampling after three months on the dietary treatments, the feeding trial was continued for another six months (nine months of dietary treatment total) using the remaining fish in each group, and then again fish weight and length were recorded, then distal intestinal samples for the histological characterization of enteritis in the experimental group was collected. Fish health, mortalities, tank densities, and tank feed conversion ratios were recorded and remained within acceptable ranges throughout the experiment (Supplementary Table S2).

### 4.2. Histological Analysis

The distal intestinal samples were removed from formalin, washed in 70% (*v*/*v*) ethanol and progressively dehydrated with increasing concentrations of ethanol. Xylene was used to clear away the ethanol, and the samples were then embedded in paraffin wax. These samples were then cut (5 µm) with a rotary microtome (HM 340E, ThermoScientific) and stained with hematoxylin and eosin. Pictures were taken with a microscope mounted with a camera (Leica DM3000 LED) at 200× magnification and imaging software was used to measure villi length and width. In addition, slides were visually scored (1—best; 5—worst) in duplicate by a blinded independent reviewer for eosinophilic granulocytes, goblet cells, inflammatory cells, lamina propria, mucosal fold fusion, subepithelial mucosa, and supranuclear vacuoles.

### 4.3. Sample Processing

Blood and fecal material were processed immediately after collection. Blood samples were centrifuged (3000× *g*, 10 min) to separate plasma. Plasma was collected and processed using commercial Amicon Ultra 0.5 mL centrifugal filters with 3 kDa MW cutoff (Millipore, UFC500396), following the manufacturer’s protocol. Filters were pre-rinsed 7 times with milliQ water and all steps were performed on ice and in refrigerated centrifuges set to 4 °C. Filtered plasma samples were stored at −80 °C until further analysis.

After collection, intestinal content samples were weighed and kept on ice during all processing steps. PBS (1×, with sodium azide) was added to the samples at a 1000:1 (*w*/*v*) ratio, and samples were homogenized by vortexing. The homogenate was incubated on ice for 10 min, vortexed, and centrifuged for 15 min at 16,000× *g* at 4 °C. The supernatant fraction was recovered and stored at −20 °C until NMR analysis.

### 4.4. NMR Acquisition

Plasma samples were mixed with phosphate buffer (1.75 M K_2_HPO_4_ (anhydrous); 1.167 mM sodium formate; 5.0 M 3-(trimethylsilyl) propionic-2,2,3,3-d_4_ acid sodium salt (TSP); pD 7.41; in ^2^H_2_O) and 99.8% ^2^H_2_O, as reference from the Bayesil online platform [42]. For each sample, a spectrum was acquired using the Nuclear Overhauser effect (tnnoesy) pulse sequence (spectral width: 7200 Hz; mixing time: 100 ms; recycle delay: 10 ms; saturation delay: 990 ms; acquisition time: 4 s; transients: 128).

Digesta samples were mixed with phosphate buffer (50 mM, pD 7.41, with 4.966 mM TSP), with sodium azide, in ^2^H_2_O) and transferred into 3 mm NMR tubes. Spectra were collected using a CPMG pulse sequence (Carr–Purcell–Meiboom–Gill sequence with the following parameters: spectral width: 7200 Hz; relaxation delay: 2 s; saturation time: 2 s; acquisition time: 3 s; 256 ecopulses; 512 ms ecotime). All spectra were acquired on a Varian VNMRS 600 MHz (Agilent, Santa Clara, CA, USA) spectrometer equipped with a 3 mm ^1^H(X)-PFG inverse configuration probe.

### 4.5. Metabolite Profiling

Identification and quantification of plasma metabolites were performed using the fully automated Bayesil online system, according to the instructions provided in the webpage (http://www.bayesil.ca, accessed on 7 July 2019 [42]. Metabolite profiling was completed selecting the custom spectral library (all metabolites) and the standard profiling speed previously described for serum [43].

Digesta NMR spectra were processed in the ACD/NMR Processor Academic Edition from ACD\Labs 12.0 software (Advanced Chemistry Development, Inc., Toronto, ON, Canada), applying zero-filling to 65 k, line broadening of 0.2 Hz, and phasing and baseline correction. TSP (singlet at 0 ppm) was used to reference chemical shifts. Metabolite identification and quantification was performed using the same software, assisted by the Reference Library version 10 of Chenomx Evaluation Edition (Chenomx Inc., Edmonton, AB, Canada), the Biological Magnetic Resonance Data Bank (http://www.bmrb.wisc.edu, accessed on 30 August 2019, and in-house databases. Metabolites were identified at Metabolomics Standards Initiative (MSI) level 2 according to the guidelines for metabolite identification [44].

### 4.6. Statistical Analyses

Fish weight was statistically assessed by fitting a two-way linear model and conducting ANOVA with a significance threshold of *p* < 0.05. When significant main effects were detected, a Tukey’s HSD test was used to differentiate pairwise group comparisons under the full model. Quantitative histological data (i.e., villi length and villi width) were analyzed by Kruskal–Wallis test to (1) determine dietary differences (FM vs. PM) within each timepoint, and (2) strain differences (ARS-*Sel*, CS-1 and CS-2) within diet and timepoint. Ordinal histological scoring data (i.e., mucosal fold fusion, goblet cell density, supranuclear vacuole density, eosiniophilic granulocyte density, inflammatory cell density, subepithelial mucosa integrity, and lamina propria structure) were analyzed by a paired Wilcoxon rank-sum test comparing both commercial strains to ARS_Sel strain within diet and timepoint, with Holm–Bonferroni multiple comparisons corrections. For the plasma and digesta metabolomics samples, metabolite concentrations were analyzed across the six experimental groups using multivariate analysis (principal component analysis (PCA), and partial least squares analysis (PLS)) conducted with MetaboAnalyst 4.0 (https://www.metaboanalyst.ca, accessed on 24 March 2021 [45]. For the PLS analysis, Q^2^ (predictive ability of the model), R^2^ (goodness of the fit), and the *p*-value of the permutation test (1000 permutations) were assessed for each model. Models were accepted as valid for Q^2^ > 0.5 and *p*-value < 0.05 [46]. All ellipses in the score plots for both PCA and PLS models were drawn at the 95% confidence level.

## 5. Conclusions

The NMR-metabolomics approach was adequate to evaluate strain variations in the metabolite profiles of samples collected by non-invasive techniques of fish fed different dietary protein sources. In the recent years, improved probe manufacturing and tailored pulse sequences (e.g., homonuclear decoupling) resulting in increased sensitivity and paved the way to low-field permanent magnet spectrometers [47,48]. Despite the high-field counterparts still providing better sensitivity and resolution, the lack of cryogenics will allow for a drastic reduction in size and operational costs, becoming an even more practical procedure for high-throughput quality control and non-lethal screening [49,50]. PLS models of blood plasma were able to separate the metabolite profiles of the selected strain from the commercial strains fed PM diets in the early stages of enteritis development. Most of the differences between metabolite profiles were observed in amino acids and could be related to modifications of intestinal physiology due to the onset of enteritis-related pathology. The higher growth performance of the ARS-*Sel* trout suggests the selected strain is better adapted to process and utilize nutrients from plant-based diets than the commercial strains evaluated herein. It was also emphasized that strain selection in rainbow trout is a suitable tool to improve this species toward better zootechnical performance without compromising fish physiology. Furthermore, our findings highlight the potential of non-invasive metabolomic samples to aid in the prediction and monitoring of the onset of soybean-meal induced enteritis.

## Figures and Tables

**Table 1:** Fish weights (g) by diet and fish strain after 3 and 9 months on treatment diets. Weights are listed as mean ± SD (n = 10). Tukey’s HSD post-hoc test was used to conducted pairwise tests under the full model when significant main effects were detected. Only significant group comparisons are listed for the strain:diet interaction post-hoc test. Significant *p*-values are italicized (NA: not analyzed).

Strain	Diet	3 Months	9 Months
ARS_*Sel*	FM	167.7 ± 31.79	936.8 ± 349.5
	PM	192.2 ± 25.51	742.8 ± 124.4
CS_1	FM	188.0 ± 29.71	872.0 ± 162.7
	PM	167.0 ± 28.69	587.9 ± 133.6
CS_2	FM	152.7 ± 15.20	1013 ± 179.0
	PM	154.7 ± 23.58	725.6 ± 126.0
Two-way ANOVA	Strain	0.004	0.078
	Diet	0.790	<0.001
	Strain:Diet	0.030	0.340
Tukey’s HSD (Strain)	ARS_*Sel* vs. CS_1	0.953	NA
	ARS_*Sel* vs. CS_2	0.007	
	CS_1 vs. CS_2	0.016	
Tukey’s HSD (Strain:Diet)	CS_1:FM vs. CS_2:FM	0.045	NA
	ARS_*Sel*:PM vs. CS_2:FM	0.017	
	CS_2:PM vs. ARS_*Sel*:PM	0.027	

**Table 2:** Summary of the results of the pairwise multivariate analysis in plasma and digesta samples for fish strains subjected to the same diet: *p*-value of the permutation testing of the PLS models, and list of metabolites assigned as VIP (score ≥ 1).

	Pairwise Comparison	PLS Permutation Test	Metabolites (VIP Score ≥ 1)	Figure
Plasma	ARS-*Sel*_PM vs. CS_PM	*p* = 0.025	alanine, glycine, arginine, lactate, betaine, lysine, sarcosine, acetoacetate, methionine, citrate, glutamine, glucose, creatine	Figure 2
	ARS-*Sel*_FM vs. CS_FM	*p* = 0.040	myo-inositol, arginine, histidine, choline, glycine, lysine, lactate, formate, proline, 3-hydroxyisobutyrate	Figure 3
Digesta	ARS-*Sel*_PM vs. CS_PM	*p* = 0.042	succinate, glucose, glycerol, valine, isoleucine, glucose-6P, sucrose, aspartate, acetate	Figure 4
	ARS-*Sel*_FM vs. CS_FM	*p* = 0.604	-	Figure S3

**Table 3:** Dietary composition and proximate analysis of the two diets administered. Values listed as mean (from technical duplicates).

Ingredient (%)	PM	FM
Soybean meal ^a^	25	--
Soy protein concentrate ^b^	23.43	--
Corn protein concentrate ^c^	10.23	--
Fish meal ^d^	--	28.2
Poultry by-product meal ^e^	--	21.52
Blood meal ^f^	--	4.3
Wheat flour ^g^	13.3	27.56
Wheat gluten meal	2.24	--
Fish oil ^h^	17	14.4
Lysine HCl	1.85	1.12
Methionine	0.59	0.42
Threonine	0.32	0.58
Taurine ^i^	0.5	--
Dicalcium phosphate	2.75	--
Vitamin premix ^j^	1	1
Choline CL	0.6	0.6
Vitamin C ^k^	0.2	0.2
Trace min premix ^l^	0.1	0.1
Potassium chloride ^m^	0.56	--
Sodium Chloride	0.28	--
Magnesium oxide ^m^	0.05	--
Calculated Composition, as-is basis		
Crude Protein, %	40	40
Crude Lipid, %	20	20
Phosphorus, %	0.9	1.16

^a^ Archer Daniels Midland Company, 472 g/kg protein; ^b^ Solae, Pro-Fine VF, 693 g/kg crude protein; ^c^ Cargill, Empyreal 75, 756 g/kg crude protein; ^d^ Menhaden Special Select, Omega Proteins Corp, 610 g/kg crude protein; ^e^ IDF Inc., 832 g/kg protein; ^f^ Wilbur-Ellis, 892 g/kg crude protein; ^g^ Manildra Milling, 120 g/kg protein; ^h^ Omega Proteins Inc., Virginia Prime menhaden oil; ^i^ NB Group Co. LTD.; ^j^ DSM Nutritional Products, ARS 702, per kg diet; vitamin A 9650 IU; vitamin D 6600 IU; vitamin E 132 IU; vitamin K3 1.1 gm: thiamin mononitrate 9.1 mg; riboflavin 9.6 mg; pyridoxine hydrochloride 13.7 mg; pantothenate DL-calcium 46.5 mg; cyanocobalamin 0.03 mg; nicotinic acid 21.8 mg; biotin 0.34 mg; folic acid 2.5 mg; inositol 600 mg.; ^k^ Stay-C, 35%, DSM Nutritional Products; ^l^ Sigma-Aldrich Company, ARS 640, contributed in mg/kg of diet; manganese 13; iodine 5; copper 9; zinc 40.; ^m^ Sigma-Aldrich Company.—Absent in the diet.

## Data Availability

The raw spectra obtained during the current study have been uploaded to the Zenodo repository (https://zenodo.org, accessed on 22 July 2021 with the reference doi:0.5281/zenodo.5121519.

## Supplementary Materials

The following are available online at https://www.mdpi.com/article/10.3390/metabo11090590/s1, Table S1: Amino acid percentage of two diets administered (wet basis). Values listed as mean from four technical replicates, Figure S1: Pairwise multivariate analysis of the CS-1 and CS-2 groups by diet, in plasma. (A) PCA scores plot of the CS-1_PM and CS-2_PM groups; (B) PLS scores plot of the CS-1_PM and CS-2_PM groups, model not validated (NC = 2; R^2^ = 0.883; Q^2^ = 0.499; 1000 permutations: *p* = 0.388); (C) PCA scores plot of the CS-1_FM and CS-2_FM groups; (D) PLS scores plot of the CS-1_FM and CS-2_FM groups, model not validated (NC = 4; R^2^ = 0.922; Q^2^ = 0.582; 1000 permutations: *p* = 0.728), Figure S2: Pairwise multivariate analysis of the CS-1 and CS-2 groups by diet, in digesta: (A) PCA scores plot of the CS-1_PM and CS-2_PM groups; (B) PLS scores plot of the CS-1_PM and CS-2_PM groups, model not validated (NC = 4; R^2^ = 0.788; Q^2^ = 0.044; 1000 permutations: *p* = 0.317); (C) PCA scores plot of the CS-1_FM and CS-2_FM groups; (D) PLS scores plot of the CS-1_FM and CS-2_FM, model not validated (NC = 1; R^2^ = 0.339; Q^2^ = 0.026; 1000 permutations: *p* = 0.127), Figure S3: Multivariate analysis of ARS_FM and CS_FM groups in digesta: (A) PCA scores plot; (B) PLS scores plot, model not validated (NC = 1; R^2^ = 0.299; Q^2^ = 0.082; 1000 permutations: *p* = 0.604).
