# Supplementary material for: Digesta and Plasma Metabolomics of Rainbow Trout Strains with Varied Tolerance of Plant-Based Diets Highlights Potential for Non-Lethal Assessments of Enteritis Development
Source: Metabolites. 2021 Sep 1;11(9):590. doi: 10.3390/metabo11090590 (PMC8470503; doi:10.3390/metabo11090590)
Supplement: Supplementary file 1 [file metabolites-11-00590-s001.zip › metabolites-1334759-supplementary.pdf]

metabolites

SUPPLEMENTARY MATERIAL SUBMITTED TO METABOLITES (ISSN: 2218-1989) Article

Digesta and Plasma Metabolomics of Rainbow Trout Strains with Varied Tolerance of Plant-Based Diets Highlights Potential for Non-Lethal Assessments of Enteritis Development

Mariana Palma [1,†], Jacob W. Bledsoe [2,†], Ludgero C. Tavares [3,4], Nicholas Romano [5], Brian C. Small [6], Ivan Viegas [1,*] and Ken Overturf [2]

[1] University of Coimbra, Centre for Functional Ecology, Department of Life Sciences, 3000-456 Coimbra, Portugal; mpalma@uc.pt

[2] ARS-USDA, Hagerman Fish Culture Experiment Station, Hagerman, ID, USA, 83332; bledsoe@uidaho.edu (J.W.B.); ken.overturf@usda.gov (K.O.)

[3] Center for Neuroscience and Cell Biology, University of Coimbra, 3004-517 Coimbra, Portugal

[4] CIVG-Vasco da Gama Research Center, University School Vasco da Gama-EUVG, 3020-210 Coimbra, Portugal; ludgero.tavares@euvg.pt

[5] Center of Excellence in Aquaculture & Fisheries Center, University of Arkansas at Pine Bluff, Pine Bluff, AR, USA, 71601; romanon@uapb.edu

[6] Aquaculture Research Institute, Hagerman Fish Culture Experiment Station, University of Idaho, Hagerman, ID, USA, 83332; bcsmall@uidaho.edu

* Correspondence: iviegas@uc.pt

† Authors contributed equally to this work.

Supplementary Table S1. Amino acid profile (%; as fed) of the two diets administered. Values listed as mean from four technical replicates.

Amino Acid (%)	PM Diet	FM Diet
Pro	2.47	2.60
Tau	0.43	0.37
Asp	1.18	1.33
Thr	2.01	2.10
Ser	4.21	4.12
Glu	3.73	3.22
Gly	1.03	0.86
Ala	2.38	2.23
Cys	1.95	2.04
Val	0.69	0.48
Met	1.07	1.06
Ile	2.05	2.27
Leu	2.36	2.71
Tyr	4.43	4.51
Phe	9.20	8.11
His	1.78	2.17
Trp	2.43	2.11
Lys	2.31	2.21
Arg	0.53	0.42

Supplementary Table S2. Growth performance data, including feed conversion ratio (FCR).

**Strain**	**Diet**	**Timepoint**	**Start #**	**Morts**	**End #**	**Tank Density (g/L)**	**Tank FCR**
ARS_*Sel*	PM	3 months	30	3	27	55,1	0,79
ARS_*Sel*	FM	3 months	30	5	25	50,4	0,98
CS_1	PM	3 months	30	1	29	49,9	1,01
CS_1	FM	3 months	30	2	28	48,9	0,80
CS_2	PM	3 months	30	2	28	51,4	0,99
CS_2	FM	3 months	30	1	29	67,2	0,78
ARS_*Sel*	PM	9 months	27	5	22	100,0	1,29
ARS_*Sel*	FM	9 months	25	0	25	108,5	1,10
CS_1	PM	9 months	29	1	28	102,1	1,42
CS_1	FM	9 months	28	5	23	95,1	1,82
CS_2	PM	9 months	28	1	27	96,5	1,73
CS_2	FM	9 months	29	3	26	131,0	1,63

Supplementary Table S3. Metabolites identified in blood plasma, average concentration (mM) and standard deviation (SD) by experimental group.

	ARS-Sel_PM		ARS-Sel_FM		CS-1_PM		CS-1_FM		CS-2_PM		CS-2_FM	
	Average	SD	Average	SD	Average	SD	Average	SD	Average	SD	Average	SD
**3-Hydroxyisobutyrate**	27.4	19.7	20.1	18.0	20.0	10.8	26.0	16.2	19.4	17.1	29.7	21.7
**Butyrate**	8.4	4.6	12.1	8.3	11.4	6.4	11.0	6.3	10.3	5.8	12.0	8.0
**Acetic acid**	6.9	0.7	7.0	1.1	7.7	2.4	6.1	1.6	7.7	1.6	6.3	1.0
**Betaine**	39.6	9.0	264.2	123.6	38.9	10.2	166.9	56.1	92.5	35.2	340.3	122.1
**Acetoacetate**	21.8	11.2	9.6	4.7	15.2	2.9	11.4	3.2	14.5	3.8	8.2	4.6
**Carnitine**	0.0	0.0	4.3	4.2	1.0	1.6	4.0	5.9	0.0	0.0	15.6	9.3
**Creatine**	65.7	23.0	109.4	60.8	90.7	50.5	100.6	42.4	35.6	9.5	89.7	44.5
**Dimethylamine**	4.1	1.9	12.8	3.3	5.5	4.1	12.4	2.7	3.8	2.2	12.3	5.1
**Citric acid**	40.3	16.3	32.4	11.7	33.8	10.6	35.7	8.9	26.6	9.4	30.3	9.3
**Choline**	11.8	1.5	11.9	5.1	12.0	2.7	17.4	4.0	17.6	2.9	17.8	7.7
**D-Glucose**	3367.2	850.7	3983.8	935.2	3738.5	1039.7	4853.9	1090.5	4293.5	1137.1	7566.3	4586.0
**Glycine**	611.4	145.1	747.2	293.5	467.0	65.8	513.6	125.4	401.8	98.1	496.6	97.0
**Formate**	108.3	15.7	87.7	18.8	113.3	20.3	97.2	9.3	114.4	14.1	105.5	14.6
**L-Glutamic acid**	129.9	33.8	102.5	34.2	118.4	50.2	75.8	21.4	117.0	30.4	99.9	19.6
**Tyrosine**	45.3	14.1	36.6	14.8	48.0	9.6	38.0	4.7	49.4	14.4	31.1	11.4
**L-Phenylalanine**	235.5	51.0	222.3	59.0	231.2	22.1	192.0	35.2	226.8	44.9	188.3	35.1
**L-Alanine**	193.4	44.5	122.1	18.2	136.6	23.5	122.3	36.4	133.4	32.2	126.2	20.5
**L-Proline**	114.5	52.7	37.9	22.0	69.7	37.3	44.7	19.0	98.2	41.5	44.6	19.4
**L-Threonine**	489.8	172.3	268.9	62.1	484.0	178.2	237.0	55.6	385.2	72.2	351.9	158.6
**L-Asparagine**	129.4	42.7	115.0	31.2	127.2	28.1	107.3	28.6	103.6	34.9	98.0	36.8
**Isoleucine**	278.1	99.2	175.9	36.7	218.6	31.1	187.3	34.5	280.5	64.5	186.1	40.0
**L-Histidine**	391.4	107.3	273.4	115.6	451.7	167.2	378.3	113.1	361.1	154.3	435.9	166.2
**L-Lysine**	234.9	55.1	127.8	26.5	159.9	41.2	99.1	34.0	191.6	59.3	102.8	28.7
**L-Lactic acid**	1683.7	310.0	2037.1	172.6	2244.6	281.3	2693.6	564.4	2019.5	448.0	2340.4	697.1
**Aspartate**	28.7	10.5	21.6	11.3	23.6	10.1	23.7	9.7	30.3	15.9	18.6	11.3
**Myo-inositol**	158.4	30.5	86.2	46.0	158.2	37.8	147.9	58.5	205.7	40.1	172.2	72.0
**Pyruvic acid**	12.1	4.6	10.0	3.8	14.1	3.0	12.7	7.9	11.6	4.5	13.7	9.1
**Taurine**	1063.4	239.1	396.1	131.3	1004.1	311.4	324.3	72.8	1379.2	334.8	530.6	261.5
**Succinate**	2.3	2.1	2.0	1.5	2.4	1.6	2.1	1.5	2.1	1.3	2.2	1.0
**Sarcosine**	20.4	5.4	34.8	11.4	14.2	2.6	32.5	6.8	16.2	3.5	40.9	9.1
**L-Arginine**	134.8	27.4	95.8	35.8	78.9	26.5	63.9	39.7	92.0	40.8	50.4	27.9
**Creatinine**	6.3	5.2	6.1	4.0	7.0	4.7	6.5	5.2	9.3	2.9	7.5	5.5
**L-Glutamine**	554.7	93.2	466.1	179.5	465.2	98.0	412.3	116.5	490.5	131.1	450.2	114.3
**L-Leucine**	554.2	201.1	319.8	56.2	415.2	74.9	337.7	61.6	550.3	138.7	357.1	70.7
**Methionine**	628.3	136.2	222.2	52.7	491.0	128.1	193.9	40.1	521.5	97.2	218.6	57.0
**Valine**	530.2	183.2	422.9	73.5	439.2	55.9	423.7	62.8	538.9	119.3	469.8	81.8

Supplementary Table S4. Metabolites identified in digesta, average concentration (mM) and standard deviation (SD) by experimental group.

	ARS-Sel_PM		ARS-Sel_FM		CS-1_PM		CS-1_FM		CS-2_PM		CS-2_FM	
	Average	SD	Average	SD	Average	SD	Average	SD	Average	SD	Average	SD
**Valine**	15.2	6.3	5.0	2.9	10.0	6.9	4.7	1.6	6.0	2.8	7.9	4.9
**Leucine**	12.8	4.9	4.3	2.0	10.0	7.8	4.4	1.5	9.4	4.9	8.4	5.0
**Isoleucine**	20.5	10.7	6.6	2.4	12.4	7.0	5.6	2.9	5.9	1.1	14.4	11.6
**Threonine Lactate**	5.6	3.2	2.3	1.2	4.0	2.8	3.0	1.3	3.0	1.6	4.4	2.3
**Alanine**	12.5	4.5	4.3	2.5	8.7	6.4	4.6	1.9	7.8	4.2	8.8	4.8
**Acetate**	10.6	9.6	2.7	2.2	5.8	3.2	1.7	0.6	2.7	0.9	2.3	1.1
**Methionine**	11.9	5.1	3.3	1.4	9.4	5.7	3.9	1.9	6.0	2.9	8.0	4.7
**Glutamate**	13.8	11.3	6.2	4.5	18.2	12.8	8.3	3.4	13.2	7.2	18.5	11.2
**Succinate**	0.2	0.3	0.0	0.0	0.5	0.4	0.1	0.1	0.3	0.3	0.2	0.2
**Aspartate**	4.3	6.3	1.6	2.0	6.2	6.9	2.7	2.6	3.8	3.1	6.4	4.7
**Creatine/Creatine-P/Creatinine**	1.0	0.9	0.7	0.6	1.1	1.0	1.3	0.7	0.4	0.3	1.7	0.9
**Dimethyl sulfone**	0.1	0.2	0.1	0.1	0.2	0.2	0.2	0.2	0.2	0.2	0.3	0.4
**Choline/Carnitine**	1.2	1.4	5.2	5.4	1.0	1.0	7.5	4.3	0.4	0.3	8.8	6.6
**Glycerol**	86.3	66.8	26.3	21.5	42.8	43.5	22.7	27.4	28.4	13.3	46.6	44.2
**Glycine**	48.4	37.5	20.0	16.2	26.8	13.2	9.7	5.1	16.6	9.6	19.1	10.0
**Glucose 6P**	0.0	0.0	0.0	0.0	0.0	0.0	0.0	0.0	1.8	1.8	0.6	0.9
**Glucose**	36.6	23.5	18.1	14.2	10.8	9.6	15.8	13.9	9.4	5.6	28.1	24.5
**Sucrose**	41.5	60.5	18.5	17.7	3.5	4.3	3.9	3.6	2.7	2.2	10.0	9.8
**Tyrosine**	1.3	2.0	0.3	0.5	1.4	1.6	0.8	0.7	1.4	1.1	0.6	0.6
**Phenylalanine**	2.5	4.5	2.0	2.5	4.4	2.5	2.0	1.7	4.1	2.7	2.8	1.3
**Formate**	0.0	0.0	0.0	0.0	0.0	0.0	0.0	0.0	0.1	0.1	0.0	0.0

Figure S1

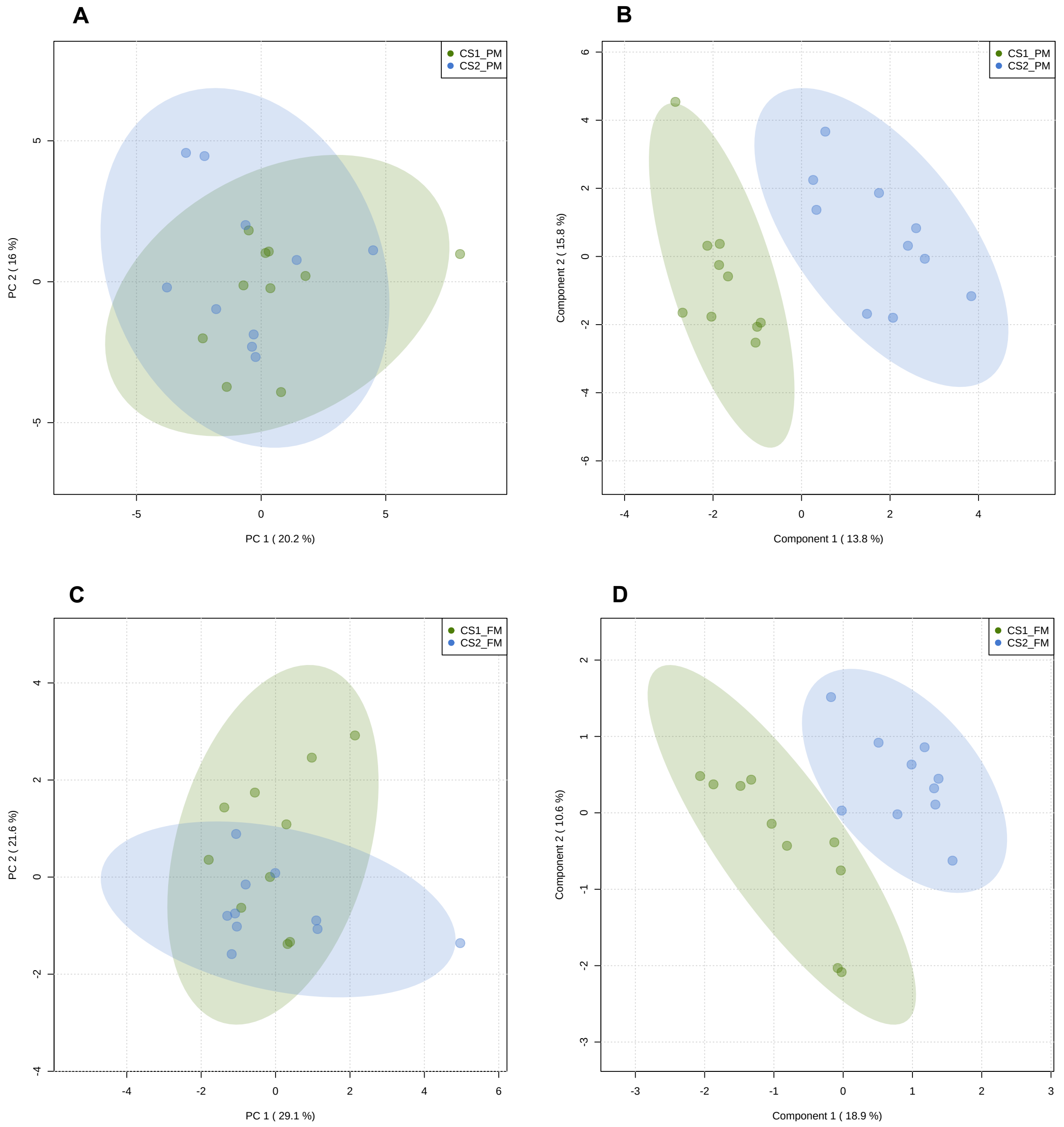

Supplementary Figure S1. Pairwise multivariate analysis of the groups CS-1 and CS-2 by diet, in plasma. **A)** PCA scores plot of the CS-1_PM and CS-2_PM groups; **B)** PLS scores plot of the CS-1_PM and CS-2_PM groups, model not validated (NC = 2; R^2 = 0.883; Q^2 = 0.499; 1000 permutations: p = 0.388); **C)** PCA scores plot of the CS-1_FM and CS-2_FM; **D)** PLS scores plot of the CS-1_FM and CS-2_FM, model not validated (NC = 4; R^2 = 0.922; Q^2 = 0.582; 1000 permutations: p = 0.728).

Figure S2

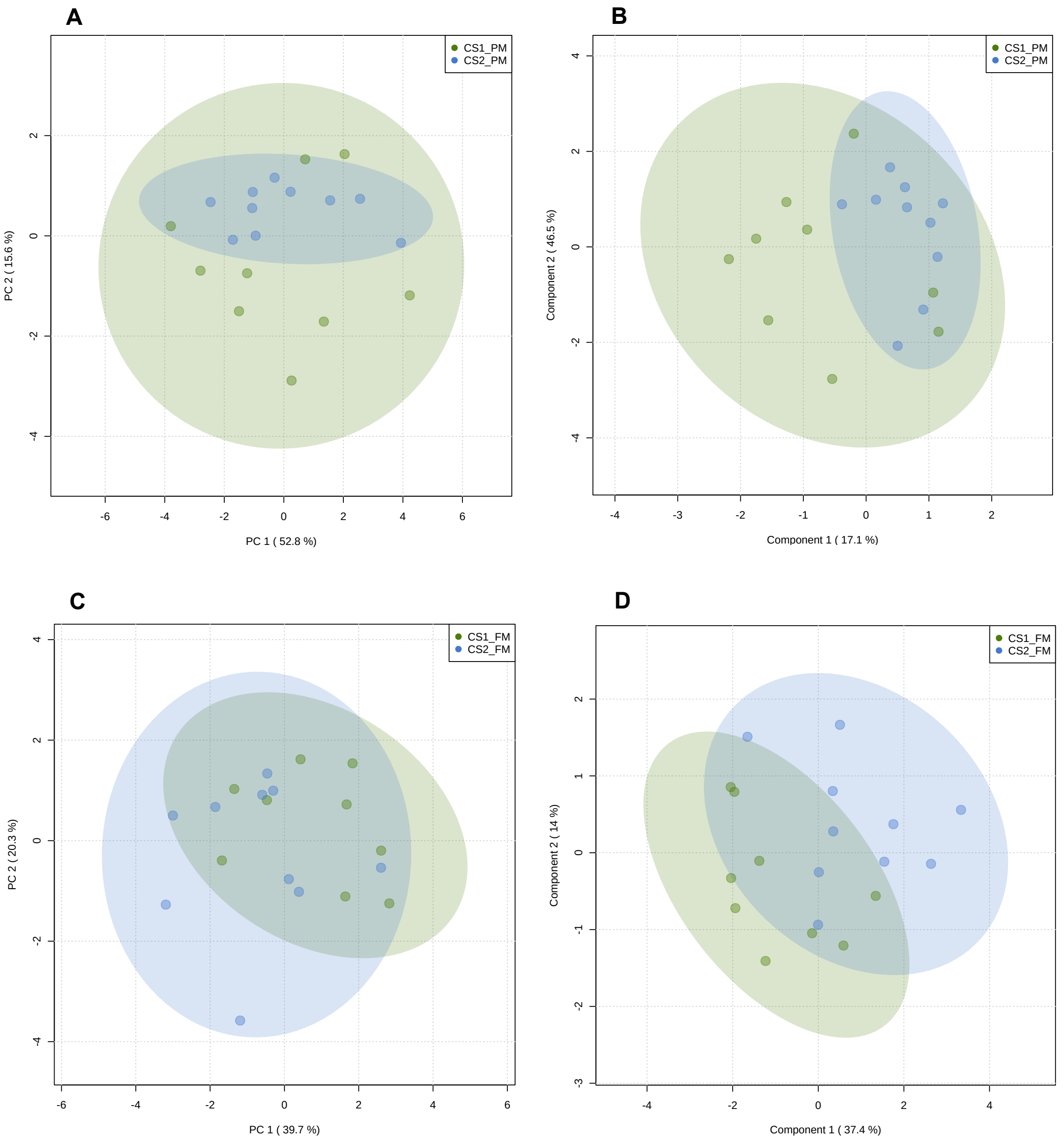

Supplementary Figure S2. Pairwise multivariate analysis of the groups CS-1 and CS-2 by diet, in digesta: **A)** PCA scores plot of the CS-1_PM and CS-2_PM; **B)** PLS scores plot of the CS-1_PM and CS-2_PM, model not validated (NC = 4; R^2 = 0.788; Q^2 = 0.044; 1000 permutations: p = 0.317); **C)** PCA scores plot of the CS-1_FM and CS-2_FM; **D)** PLS scores plot of the CS-1_FM and CS-2_FM, model not validated (NC = 1; R^2 = 0.339; Q^2 = 0.026; 1000 permutations: p = 0.127).

Figure S3

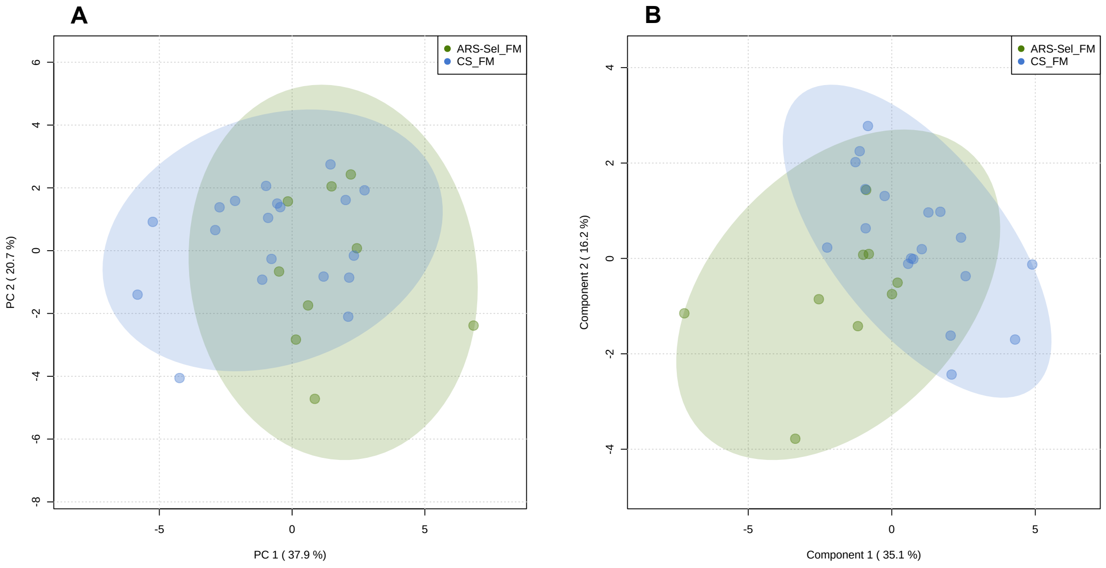

Supplementary Figure S3. Multivariate analysis of groups ARS_FM and CS_FM in digesta: **A)** PCA scores plot; **B)** PLS scores plot, model not validated (NC = 1; R^2 = 0.299; Q^2= 0.082; 1000 permutations: p = 0.604).
